# ASGARD is A Single-cell Guided Pipeline to Aid Repurposing of Drugs

**DOI:** 10.1038/s41467-023-36637-3

**Published:** 2023-02-22

**Authors:** Bing He, Yao Xiao, Haodong Liang, Qianhui Huang, Yuheng Du, Yijun Li, David Garmire, Duxin Sun, Lana X. Garmire

**Affiliations:** 1 Department of Computational Medicine and Bioinformatics, Medical School, University of Michigan, Ann Arbor, MI USA; 2 Department of Statistics, College of Literature, Science, and the Arts, University of Michigan, Ann Arbor, MI USA; 3 Department of Electrical Engineering and Computer Science, College of Engineering, University of Michigan, Ann Arbor, MI USA; 4 Department of Pharmaceutical Sciences, College of Pharmacy, University of Michigan, Ann Arbor, MI USA

**Keywords:** Computational biology and bioinformatics, Drug discovery, Drug development, Translational research, Computational science

## Abstract

Single-cell RNA sequencing technology has enabled in-depth analysis of intercellular heterogeneity in various diseases. However, its full potential for precision medicine has yet to be reached. Towards this, we propose A Single-cell Guided Pipeline to Aid Repurposing of Drugs (ASGARD) that defines a drug score to recommend drugs by considering all cell clusters to address the intercellular heterogeneity within each patient. ASGARD shows significantly better average accuracy on single-drug therapy compared to two bulk-cell-based drug repurposing methods. We also demonstrated that it performs considerably better than other cell cluster-level predicting methods. In addition, we validate ASGARD using the drug response prediction method TRANSACT with Triple-Negative-Breast-Cancer patient samples. We find that many top-ranked drugs are either approved by the Food and Drug Administration or in clinical trials treating corresponding diseases. In conclusion, ASGARD is a promising drug repurposing recommendation tool guided by single-cell RNA-seq for personalized medicine. ASGARD is free for educational use at https://github.com/lanagarmire/ASGARD.

## Abstract (web summary)

The full potential of single-cell RNA-sequencing applied to precision medicine has yet to be reached. Here, we propose a drug recommendation system ASGARD, which predicts drugs by considering cell clusters to address the intercellular heterogeneity within each patient.

## Introduction

Heterogeneity, or more specifically, the diverse cell populations within the diseased tissue, is the leading cause of treatment failure for many complex diseases, such as cancers^1^, Alzheimer’s disease^2^, stroke^3^, and coronavirus disease 2019 (COVID-19)^4^, etc., as well as a major obstacle to successful precision medicine^5–7^. Recent significant advances in single-cell technologies, especially the single-cell RNA sequencing (scRNA-seq) technology, have enabled the analysis of intercellular heterogeneity at a very fine resolution^8,9^ and helped us to have many breakthroughs in understanding disease mechanisms^10^, such as breast cancer^11^, liver cancer^12^ and COVID-19^13^. However, its full potential for precision medicine has not been fulfilled^14,15^.

Drug repurposing (also known as drug reposition, reprofiling, or re-tasking) is a strategy to identify new drug uses outside the scope of its original medical approval or investigation^16^. So far, few drug repurposing methods have been developed to utilize the highly valuable information contained in scRNA-seq data. The pipeline by Alakwaa identifies significantly differentiated genes (DEGs) for a specific group of cells, then predicts candidate drugs for DEGs using the Connectivity Map Linked User Environment (CLUE) platform, followed by prioritizing these drugs using a comprehensive ranking score system^17^. This pipeline identified didanosine as a potential treatment for COVID-19 using scRNA-seq data^17^. Another pipeline by Guo et al. uses a simple combination of Seurat^18^, a tool for scRNA-seq analysis, and CLUE to identify 281 FDA-approved drugs that can potentially be effective for treating COVID-19^19^. In general, the above pipelines predict drugs for each cell cluster within the patient. However, in heterogeneous diseases caused by multiple types of cells, efficient drugs should be able to address multiple cell clusters^20^. Neither of these pipelines mentioned above can predict drugs for multiple cell clusters, limiting their utility in the era of precision medicine.

Here we propose A Single-cell Guided Pipeline to Aid Repurposing of Drugs (ASGARD) to overcome the issue above. ASGARD defines a drug score to predict drugs for multiple diseased cell clusters within each patient. The benchmarking results show that the performance of ASGARD on single drugs is more accurate and robust than other pipelines handling bulk and single-cell RNA-Seq data. We tested ASGARD on multiple cancer scRNA-Seq datasets, including patient-Derived Xenografts (PDXs) models for advanced metastatic breast cancers, Pre-T acute lymphoblastic leukemia patients, and primary tumors of Triple-Negative-Breast-Cancer (TNBC) patients. Additionally, with the ongoing worldwide COVID-19 pandemic, we applied ASGARD to scRNA-seq data from severe COVID-19 patients and predicted potential therapies to reduce deaths of severe COVID-19 patients.

## Results

### Summary of a Single-cell Guided Pipeline to Aid Repurposing of Drugs

**Fig. 1:**
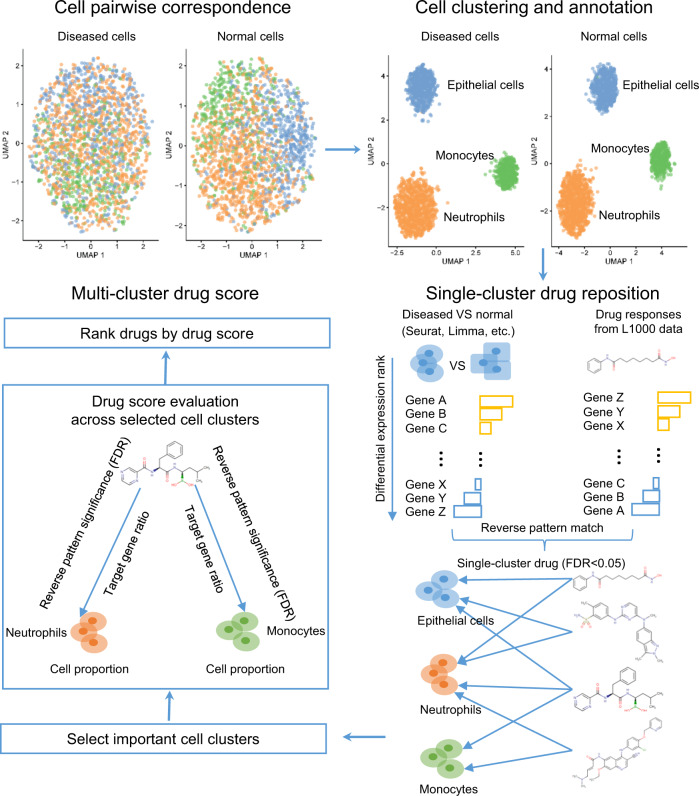
The workflow of the ASGARD drug repurposing pipeline. The workflow of the ASGARD pipeline. Diseased and normal cells are paired according to “anchor” genes that are expressed consistently between the two types of cells. The differentially expressed (DE) genes are identified between diseased and normal cells, either within a cluster or within a cell type. Using the consistent DE genes as the input, potential drugs that significantly reverse the pattern of DE genes are identified, using the Kolmogorov–Smirnov (K-S) test with Benjamini–Hochberg (BH) false discovery rate (FDR) adjustment. Next ASGARD estimates and ranks the drug scores for single drugs, by targeting specific cell cluster(s) or all cell clusters.

Using scRNA-seq data, ASGARD repurposes drugs for disease by fully accounting for the cellular heterogeneity of patients (Fig. 1, Formula 1 in “Methods” section). In ASGARD, every cell cluster in the diseased sample is paired to that in the normal (or control) sample, according to “anchor” genes that are consistently expressed between diseased and normal cells. It then imports differentially expressed (DE) genes (adjusted *P*-value < 0.05) between the paired diseased and normal clusters in the scRNA-seq data, as determined by a DE detection method at the user’s choice. These individual clusters can be optionally annotated to specific cell types. To identify drugs for each single cluster (cell type), then ASGARD uses these consistently differentially expressed genes as inputs to identify drugs that can significantly (single-cluster FDR < 0.05) reverse their expression levels in the L1000 drug response dataset^21^. To identify drugs for multiple clusters, ASGARD defines a drug score (Formula 1 in “Methods” section) to evaluate the drug efficacy across multiple cell clusters selected by the user. The drug score estimates drug efficacy by taking into account the cell type proportion, the significance of reversing the differential gene expression pattern (single-cluster FDR) by the drug treatment in each selected cell cluster, and the ratio of significantly deregulated genes (adjusted *P*-value < 0.05) that the drug treatment can reverse in each selected cell cluster. Finally, ASGARD uses the drug score to rank and choose drugs for the disease.

We evaluated the power of the drug score by comparing ASGARD with traditional bulk-cell-based repurposing methods and single-cell-based repurposing methods using multiple independent scRNA-seq datasets, including PDX models from advanced metastatic triple-negative breast cancer (TNBC)^11^, an acute lymphoblastic leukemia dataset^22^, and coronavirus disease 2019 (COVID-19) datasets^13,23^ (see “Methods” section).

### Comparing ASGARD to bulk-cell based repurposing methods

**Fig. 2:**
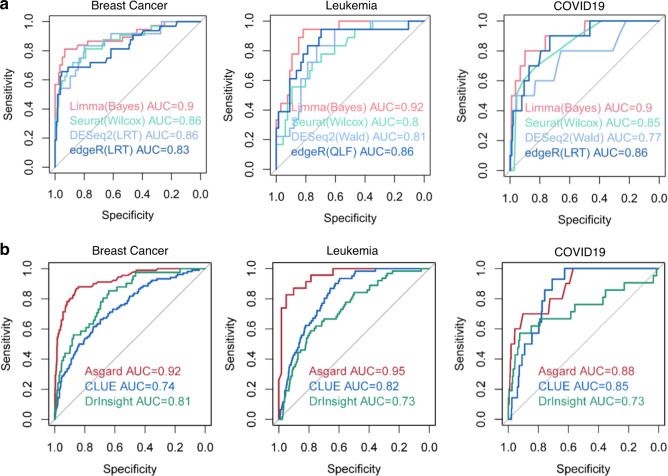
Comparing ASGARD to bulk-cell-based repurposing methods. The receiver operating characteristic (ROC) curves and area under curve (AUC) scores of the ASGARD, on advanced metastatic breast cancer, acute lymphoblastic leukemia, and coronavirus disease 2019 (COVID-19), respectively. **a** Comparison among different DE analysis methods Limma (red), DESeq2 (light blue), Seurat (green), and edgeR (blue) using the best-performing mode in each. **b** Comparison of ASGARD (red) and bulk-sample based drug repurposing methods: CLUE (blue), and DrInsight (green), using the same three diseases as in **a**. The single-cell RNA-Seq data were aggregated to pseudo-bulk RNA-Seq data as the input of the bulk-sample based methods. Source data are provided as a Source Data file.

Before comparing ASGARD to bulk-cell-based repurposing methods, we first evaluated several external differential expression (DE) methods, on three datasets from three diseases: advanced metastatic breast cancer^11,24^, acute lymphoblastic leukemia^22^, and coronavirus disease 2019^13,23^ (see “Methods” section). We selected Limma^25^, Seurat^18^, DESeq2^26^, and edgeR^27^ for DE methods, given that they were top-ranked methods in a benchmark study of confronting false discoveries in single-cell differential expression^28^. We conducted systematic comparison of these methods under different modes (Fig. 2). For Limma, we compared three modes: empirical Bayes without trend (Bayes), empirical Bayes approach prior trend (trend), and precision weights (voom)^25^. For Seurat, we compared three different DE tests: Wilcoxon rank-sum test (Wilcox), *t*-test, and logistic regression (LR). For DESeq2, we compared the Wald test (Wald) and the likelihood ratio test (LRT). For edgeR, we compared the likelihood ratio test (LRT) and the quasi-likelihood F-test (QLF). We identified DE genes using the above methods for each cell cluster as the inputs of ASGARD for drug repurposing. The subsequent drug prediction accuracies by ASGARD are determined by the receiver operating characteristic curves (ROCs) and the areas under the ROC curves (AUCs), using FDA-approved drugs and candidate drugs validated in advanced clinical trials as positive data (see “Methods” section).

The systematic comparison is shown in Supplementary Fig. 1, and the results from each method under the best-performing mode are shown in Fig. 2a. The Limma (Bayes) method yields the best AUC in all three datasets ranging from (0.90-0.92), significantly (*P*-value < 0.05, Student’s *t* test) better than other DE methods. Seurat (Wilcox test) and edgeR perform similarly overall, where edgeR has slightly higher AUC (0.83–0.86) than Seurat (0.80–8.86). DEseq2 on the other hand, tends to generate some of the lowest AUCs in comparison. Therefore, we used DE results from the Limma-Bayes package for the following analysis, while keeping other DE methods as options for the inputs to ASGARD.

To compare ASGARD with those drug repurposing methods using bulk RNA-Seq samples, we summarized scRNA-seq data into pseudo-bulk RNA-Seq data. We then applied bulk methods CLUE^29^ and DrInsight^30^ to the pseudo-bulk RNA-Seq query data and compared their results with ASGARD’s on predicting both drugs and compounds (Fig. 2b). We used the same scRNA-seq data from the same three datasets above. Since CLUE and DrInsight predict both drugs and compounds, we added compounds validated in animal models to the true positive dataset for the AUC evaluation of drug/compound predictions. As a result, the AUCs obtained from ASGARD on drugs and compounds (Fig. 2b) are slightly different from those on drugs only (Fig. 2a). On the breast cancer dataset, ASGARD yields an overall AUC of 0.92, much better than CLUE and DrInsight, with values of 0.74 and 0.81, respectively. On precursor T cell acute lymphoblastic leukemia data, ASGARD yields an AUC of 0.95 in drug/compound repurposing for leukemia patients, while CLUE and DrInsight achieve worse average AUCs of 0.82 and 0.73, respectively. For the COVID-19 datasets, ASGARD shows an AUC of 0.88 in drug/compound repurposing, while CLUE and DrInsight have lower AUCs of 0.85 and 0.73, respectively, for the same patients (Fig. 2b). In summary, by paying attention to heterogeneity at single-cell levels, ASGARD shows much better drug repurposing predictability than methods that rely on bulk samples.

### Comparing ASGARD to other single-cell-based repurposing methods

**Fig. 3:**
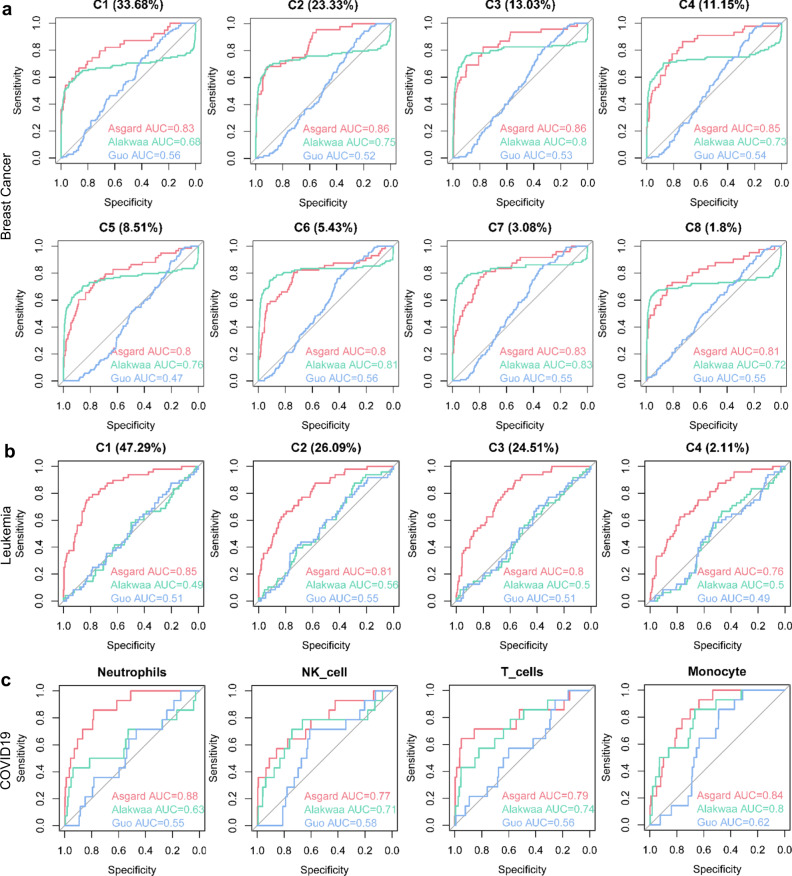
Comparing ASGARD to other single-cell-based repurposing methods. The receiver operating characteristic (ROC) curves and area under curve (AUC) scores of the ASGARD (red) and other published pipelines: Alakwaa’s pipeline (green) and Guo’s pipeline (blue). The results of drug/compound repurposing are shown on every cell cluster of the metastatic breast cancer dataset (**a**), every cell cluster of the acute lymphoblastic leukemia dataset (**b**) and 4 clusters with increased cell proportions in the decreased severe vs cured severe COVID-19 patients (**c**). The proportion of each single-cell cluster is shown in the brackets above each plot. Source data are provided as a Source Data file.

We also compared single drug prediction using ASGARD with two other pipelines developed by Alakwaa et al.^17,19^ and Guo et al.^17,19^, which were reported to handle scRNA-Seq data. Note that ASGARD offers more functionalities than those two methods. Alakwaa’ and Guo’ pipelines can only repurpose drug/compounds for every cluster, but not on a multi-cluster level. On the other hand, ASGARD can compute both the single-cluster-level drug significance and the multi-cluster drug score (Formula 1 in “Methods” section). The above section shows that the ASGARD multi-cluster drug score shows AUCs of 0.92, 0.95, and 0.88 for breast cancer, leukemia, and COVID-19, respectively (Fig. 2b). For a fair comparison, we further tested the single-cluster-level drug prediction accuracies of these three methods (Fig. 3 and Supplementary Fig. 2). Even at the single-cluster-level, ASGARD still shows the best AUCs on every individual cluster from breast cancer, leukemia, and COVID-19 datasets (Fig. 3). On the 8 clusters of the breast cancer dataset, ASGARD yields an averaged AUC of 0.83 (0.80–0.86), significantly better (*P*-value = 0.0028, Student’s *t* test) than Alakwaa’ and Guo’ pipelines, with averaged AUC values of 0.76 (0.68–0.83) and 0.54 (0.47–0.56) respectively (Fig. 3a and Supplementary Fig. 2). On the 4 clusters of precursor T cell acute lymphoblastic leukemia data, ASGARD has an averaged AUC of 0.81 (0.76–0.85), again significantly better (*P*-value < 0.001, Student’s *t* test) than Alakwaa’ and Guo’ pipelines, with averaged AUC values of 0.51 (0.49-0.56) and 0.52 (0.49-0.55) respectively (Fig. 3b and Supplementary Fig. 2). Similar trends exist in the neutrophile, NK, T cell and monocytes that have increased cell proportions in the decreased severe vs. cured severe COVID-19 patients. While ASGARD achieves average AUCs of 0.82 (0.77–0.88), Alakwaa’ and Guo’ methods have reduced average AUCs of 0.72 (0.63–0.80), and 0.58 (0.55–0.62; Fig. 3c and Supplementary Fig. 2). These results support the conclusion that ASGARD predicts drugs more accurately than Alakwaa’ and Guo’ pipelines.

Additionally, given that sample size, cell population similarity, and proportion of disease cells impact significantly on differential gene analysis^31^, we further performed robustness assessments of the three pipelines across different sizes of single-cell populations, different differential levels of single-cell populations, and different proportions of diseased cells using simulation data based on “GSE123926” and “GSE113197” dataset (see “Methods” section). The AUCs of the three single-cell drug repurposing pipelines on the simulation data show that ASGARD, as well as the other two pipelines, have very robust performance across different sizes of single-cell populations (Supplementary Fig. 3A), different degrees of DE between disease and normal conditions (Supplementary Fig. 3B), and different proportions of disease cells among the scRNA-Seq data (Supplementary Fig. 3C).

We demonstrate that ASGARD is a promising drug recommendation pipeline through computational and clinical validation. In the following sections, we further illustrate the results of ASGARD applied to breast cancer, leukemia, and COVID-19, respectively.

### Drug repurposing for breast cancers

We collected scRNA-seq data from 24,741 epithelial cells of advanced metastatic breast cancer Patient-Derived Xenografts (PDXs) models^11^ and 16,998 epithelial cells from normal breast tissues^24^. After preprocessing, all cancer cells and 16,954 normal cells were paired and clustered into 8 populations (Supplementary Fig. 4A). Cluster 1 (C1) is the largest one covering 33.68% of cells, while cluster 8 (C8) is the smallest one accounting for only 1.8% of cells (Supplementary Fig. 4A). The differentially expressed genes (adjusted *P*-value < 0.05, cancer vs normal) in the clusters are significantly enriched in 10 well-known breast cancer-related pathways, including apoptosis, cell cycle, estrogen signaling, IL − 17 signaling, neurotrophin signaling, NF − kappa B signaling, NOD − like receptor signaling, p53 signaling, PI3K − Akt signaling and TNF signaling pathways (Supplementary Fig. 4B). Cluster 7 (C7) has the largest number of 7 significant pathways, while C1 and C6 each have only 1 significant pathway.

We first applied ASGARD for multi-cluster drug repurposing prediction and predicted 11 drugs (FDR < 0.05 and overall drug score >0.99 quantiles) for advanced metastatic breast cancer (Supplementary Fig. 4C and Supplementary Data 1). Fostamatinib is the top 1 drug candidate (Supplementary Fig. 4C). It is a tyrosine kinase inhibitor medication approved for the treatment of chronic immune thrombocytopenia^32^. Colchicine, the second-best candidate, is an alkaloid approved for treating the inflammatory symptoms of familial Mediterranean fever^33^. Both fostamatinib and colchicine have shown antitumor and anti-metastasis effects in animal models of breast cancer^34,35^. Moreover, the 4th candidate fulvestrant, and 7th candidate neratinib have been approved by the Food and Drug Administration (FDA) for breast cancer treatment^36,37^.

To explore the potential molecular mechanisms of the top 2 candidates, we next investigated the target genes and pathways of fostamatinib and colchicine across the eight cell clusters (Supplementary Fig. 4D). Fostamatinib and colchicine both target all the significant pathways in each cluster. Fostamatinib and colchicine are complementary in targeting genes of these pathways. Among the 143 target genes from these significant pathways, only 29 target genes are shared by fostamatinib and colchicine (Supplementary Fig. 4D). The fostamatinib and colchicine also show biologically synergistic targeting of multiple genes on the same significant pathways. For example, fostamatinib inhibits Cyclin D1 (*CCND1*) to produce G1 arrest in the p53 signaling pathway, while colchicine inhibits Cyclin-dependent kinase 1 (*CDK1*) to produce G2 arrest in the p53 signaling pathway and cell cycle pathway^38^ (Supplementary Fig. 4D). Additionally, the drug scores of top drug candidates vary from one PDX model to another (Supplementary Fig. 4D), demonstrating that ASGARD is a forward-looking precision medicine strategy in silico.

**Fig. 4:**
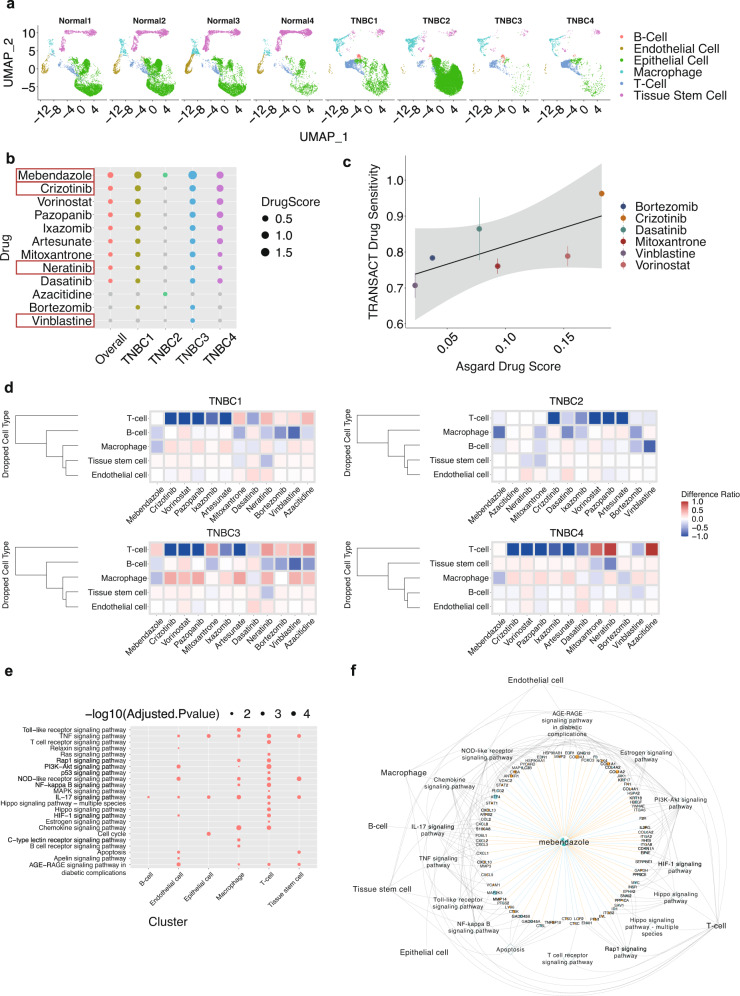
Drug repurposing in triple-negative breast cancer (TNBC) patient samples. **a** UMAP plots of single-cell data from four TNBC patient samples and four control samples from the same study. **b** The overall drug scores combining all TNBC samples as well as drug scores in each TNBC sample, among the top-ranked significant single drugs (FDR < 0.05). Gray color indicates a lack of significance for a particular drug in an individual. The four drugs in the red boxes overlapped with the top drugs predicted by ASGARD using PDX models of TNBC patients. **c** Comparison between TRANSACT drug sensitivity and ASGARD drug score. Data are presented as mean values ± /SD. *n* = 4 biologically independent samples. **d** Heatmap of the drug score changes in the cell-type-specific drop-one-out experiment, where each cell type in the tumor microenvironment is removed at a time. **e** Pathway enrichment analysis (TNBC vs. normal) for each cell cluster. *P*-values were determined by two-sided Fisher’s exact test and were adjusted by BH FDR. **f** The top drug candidate mebendazole, its target genes, pathways, and single-cell clusters. Orange node: up-regulated gene (logFC>1 and adjusted *P*-value < 0.05). Blue node: down-regulated gene (logFC < −1 and adjusted *P*-value < 0.05). Orange solid edge: drug stimulates gene expression. Blue solid edge: drug inhibits gene expression. The width of the edge is proportional to the strength of the drug effect. Gray dotted edge: gene belonging to a pathway. Gray backward slash: pathway significant in a cell cluster. *P*-values were determined by two-sided Fisher’s exact test and were adjusted by BH FDR. Source data are provided as a Source Data file.

To evaluate the reliability of ASGARD on breast cancer patient data, we downloaded four Triple Negative Breast Cancer (TNBC) samples along with four controls from the “GSE161529” dataset^39^, in order to compare with the drug prediction results from the PDX models of TNBC described earlier. After the preprocessing procedure by Seurat, the TNBC samples contain an average of 5580 cells. We aligned all 8 samples, paired the cases vs. controls, and clustered them into 6 groups: B-cell, endothelial cell, epithelial cell, macrophage, T cell, and tissue stem cell (Fig. 4a). Epithelial cells are the largest group covering 45.98% of cells on average, while endothelial cells are the smallest group as expected, accounting for only 1.152% of total cells (Fig. 4a). ASGARD predicted 13 drugs with significant FDR *p*-values in at least one of the four TNBC patients (Fig. 4b). For comparison, we also performed drug predictions on the 2 PDX models of TNBC patients, using the same procedures (Supplementary Fig. 4c). Of great interest, four of the most significant drugs from TNBC patients overlap with those predicted by the two PDX samples. These drugs are mebendazole, crizotinib, neratinib, and vinblastine (Fig. 4b). Both neratinib and vinblastine have been proven by the FDA for the treatment of breast cancer^40,41^. Mebendazole is a well-known anti-helminthic drug with wide clinical use. It has been reported to have anti-cancer properties in preclinical studies and has been in many clinical trial studies for treating various cancers, including liver, lung cancers, and glioma^42^. Crizotinib is a receptor tyrosine kinase inhibitor showing tumor-reducing effects in vitro and in vivo^43,44^. It is now in a phase 2 clinical trial for treating patients with TNBC (ClinicalTrials.gov Identifier: NCT03620643).

To show quantitatively that ASGARD prediction on the TNBC samples is valuable, we next conducted two additional sets of analyses. First, we compared its results with those using TRANSACT^45^, another computational method to calculate drug sensitivity. Since the TRANSACT can only predict drugs existing in the GDSC dataset^46^, thus we can only compare the subset of drugs predicted by ASGARD in the GDSC dataset. As shown in Fig. 4c, ASGARD and TRANSACT results are well correlated. As ASGARD’s drug score increases, the drug sensitivity in TRANSACT also increases. Second, we investigate the effect of the tumor microenvironment on the drug scores. Thus, we did in silico drop-one-out experiment, which excluded one cell type at a time. Among all cell types in the tumor microenvironment, T cell leads to the most drastic drug score changes, as well as the most variable drug score changes among different drugs (Fig. 4d). Moreover, the drug score changes also differ among the four TNBC patients, showing the sensitivity of ASGARD in personalized drug prediction.

To explore the potential molecular mechanisms of the top drug candidate, we next investigated the target genes and pathways of mebendazole across the six cell clusters (Fig. 4e, f). mebendazole targets many important genes and pathways in TNBC, such as signal transducer and activator of transcription 1 (STAT1) in Toll-like receptor signaling pathway, Vascular Cell Adhesion Molecule 1 (*VCAM1*) in NF-kappa B signaling pathway, Matrix Metallopeptidase 14 (*MMP14*) in TNF signaling pathway, signal transducer and activator of transcription 2 (*STAT2*) in NOD-like receptor signaling pathway, cyclin-dependent kinase inhibitor 1 A (*CDKN1A*) in PI3K-Akt signaling pathway, etc. These targeted genes and pathways are essential for the proliferation, migration, and invasion of TNBC cells^47,48^, and were suggested as therapeutic drug targets for TNBC in previous studies^49,50^.

### Drug repurposing for precursor T cell acute lymphoblastic leukemia (Pre-T ALL)

**Fig. 5:**
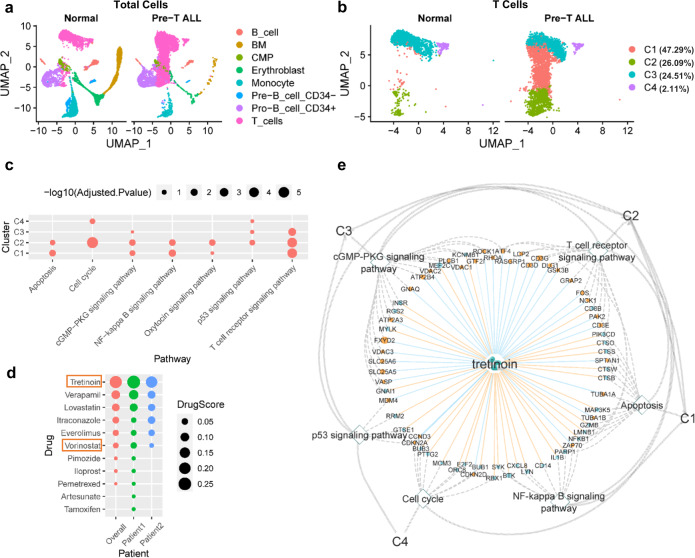
Drug repurposing for precursor T cell acute lymphoblastic leukemia (Pre-T ALL). **a** UMAP plots of all cells from 3 normal controls and 2 Pre-T ALL samples. **b** UMAP plots of T cell clusters from normal controls and Pre-T ALL samples. **c** Pathway enrichment analysis (leukemia vs normal) for each T cell cluster. *P*-values were determined by two-sided Fisher’s exact test and were adjusted by BH FDR. **d** The overall drug score and drug score in each Pre-T ALL patient, among top-ranked significant drugs (FDR < 0.05). Drug approved for leukemia treatment by the FDA is tretinoin. **e** The drug candidate tretinoin, its target genes, pathways, and single-cell clusters. All labels and their annotations are the same as Fig. 4f. *P*-values were determined by two-sided Fisher’s exact test and were adjusted by BH FDR. Source data are provided as a Source Data file.

We further applied ASGARD to the collected scRNA-seq data from 2 Pre-T ALL patients and three normal healthy controls^22^. ASGARD identifies eight types of cells (Fig. 5a), in which T cells are further clustered into four sub-populations (Fig. 5b). Cluster 1 (C1) is the largest one, covering 47.29% of cells, while cluster 4 (C4) is the smallest, accounting for only 2.11% of cells (Fig. 5b). The differentially expressed genes (adjusted *P*-value < 0.05, Pre-T ALL vs. normal) in the T cell clusters are significantly enriched in 6 pathways, including apoptosis, cell cycle, cGMP−PKG signaling, NF − kappa B signaling, p53 signaling, and T cell receptor signaling pathways (Fig. 5c).

Among the predicted drugs by ASGARD, the first candidate, tretinoin, has been approved for the treatment of leukemia^51^ (Fig. 5d and Supplementary Data 2). Tretinoin is a vitamin A derivative. We further explored the potential molecular mechanisms of the FDA-approved top1 candidate tretinoin. Tretinoin targets many leukemia-related genes and all the significant pathways in the 4T cell clusters, including: the regulator *MDM4* in the p53 signaling pathway, cyclin D3 (*CCND3*) in cell cycle and p53 signaling pathways, G protein subunit alpha q (*GNAQ*) and phospholipase C beta 1 (*PLCB1*) in the cGMP−PKG signaling pathway, Fos protooncogene (*FOS*) and p21 (RAC1) activated kinase 2 (*PAK2*) in the T cell receptor signaling pathway, spectrin alpha non-erythrocytic 1 (*SPTAN1*) in the apoptosis pathway, and zeta chain of T cell receptor-associated protein kinase 70 (*ZAP70*) in apoptosis and NF − kappa B signaling pathways (Fig. 5e). All these genes and pathways were previously shown to have significance in the pathogenesis of Pre-T ALL^52–54^. The drug target genes and pathways in the T cell clusters explain why ASGARD predicts tretinoin for leukemia and how tretinoin treats leukemia.

### Drug repurposing for severe patients with coronavirus disease 2019 (COVID-19)

**Fig. 6:**
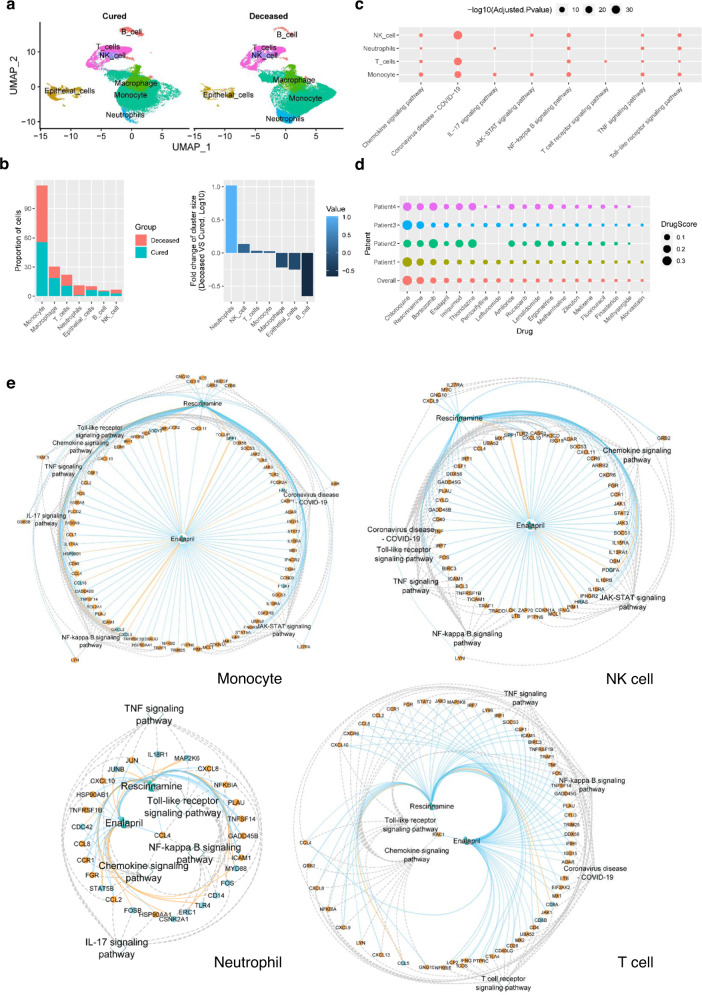
Drug repurposing for reducing mortality of severe COVID-19 patients. **a** Single-cell populations of bronchoalveolar immune cells in 11 cured and four deceased severe COVID-19 patients, respectively. **b** The proportions of cell type in (left) and log10 transformed fold changes in deceased over the cured state (right) of the single-cell populations in **a**. **c** Pathway enrichment analysis (deceased severe vs. cured severe) for neutrophil, NK cell, T cell, and monocyte, respectively. *P*-values were determined by two-sided Fisher’s exact test and were adjusted by BH FDR. **d** The drug scores in the four deceased severe COVID-19 patients and all four patients among top-ranked significant drugs (FDR < 0.05). **e** The drug candidate rescinnamine and enalapril, their target genes and pathways in the monocyte, NK cell, T cell, and neutrophil, respectively, from severe COVID-19 patients. All labels and their annotations are the same as in Fig. 4f. *P*-values were determined by two-sided Fisher’s exact test and were adjusted by BH FDR. Source data are provided as a Source Data file.

The immune response activated by the SARS-CoV-2 virus infection is a double-edged sword. It protects the human body from viral infection. But the deregulated immune response in severe COVID-19 patients damages the alveolar to cause respiratory failure that kills the patients^55,56^. To find drugs that may help to reduce the mortality of severe COVID-19, we collected scRNA-seq data from the bronchoalveolar lavage fluid (BALF) of 15 severe COVID-19 patients^13,23^. Among them, 11 patients were cured (cured severe patient), while four died (deceased severe patient) afterward. To identify immune cells that correlate with the death of severe patients, we compared the scRNA-seq data between deceased severe and cured severe patients. In total, there are seven types of cells, including six types of immune cells and epithelial cell types (Fig. 6a), in the BALF samples collected from severe COVID-19 patients. Monocyte is the largest T cell population in both deceased and cured severe COVID-19 patients (Fig. 6b). The population of neutrophil, NK cell, T cell, and monocyte increased in deceased severe COVID patients compared to the cured ones, suggesting the important role of these four types of cells in COVID-19-related death^57–60^ (Fig. 6b). The differentially expressed genes (adjusted *P*-value < 0.05, deceased severe vs cured severe) in the four types of cells are significantly enriched (adjusted *P*-value < 0.05) in 8 pathways, including chemokine signaling, coronavirus disease−COVID − 19, IL − 17 signaling, JAK − STAT signaling, NF − kappa B signaling, T cell receptor signaling, TNF signaling and Toll−like receptor signaling pathways (Fig. 6c). Coronavirus disease−COVID − 19 pathway is the most significant pathway in these cells, as expected. Chemokine signaling, NF − kappa B signaling, TNF signaling, and Toll−like receptor signaling pathways are the most widely enriched pathways in all four types of cells. T cell receptor signaling pathway is only enriched in T cells.

We identified the differential gene expression profiles of the four cell types, including neutrophil, NK cell, T cell, and monocyte, by comparing decreased severe patients to cured severe ones. Then we put the differential gene expression profiles to ASGARD to identify drug candidates using the multi-cluster drug score. Among the predicted drugs, rescinnamine (2nd) and enalapril (4th) caught our attention (Fig. 6d, Supplementary Data 3). Both rescinnamine and enalapril are angiotensin-converting enzyme (*ACE*) inhibitors. Angiotensin-converting enzyme 2 (*ACE2*) mediates the SARS-CoV-2 cell entry. It’s interesting to see rescinnamine and enalapril are predicted by ASGARD for treating severe COVID-19. So, we further explored their target genes and pathways in the four cell types. Rescinnamine and enalapril share most of the key genes on all the significant pathways in monocyte, NK cell, neutrophil, and T cell, respectively (Fig. 6e). In monocyte, rescinnamine and enalapril share 47 key target genes, including Janus Kinase 1 (*JAK1*), Janus Kinase 2 (*JAK2*), C-C Motif Chemokine Ligand 2 (*CCL2*), C-C Motif Chemokine Ligand 4 (*CCL4*), and C-C Motif Chemokine Ligand 8 (*CCL8*), and all the 7 significant pathways. In NK cells, rescinnamine and enalapril share 35 key target genes from 6 significant pathways, such as *JAK1*, Janus Kinase 3 (*JAK3*), *CCL4*, tumor necrosis factor (*TNF*), and Signal Transducer and Activator of Transcription 2 (*STAT2*). In neutrophils, rescinnamine and enalapril share 16 key target genes, such as *CCL2*, *CCL8*, C-X-C Motif Chemokine Ligand 8 (*CXCL8*) and C-X-C Motif Chemokine Ligand 10 (*CXCL10*), and all the 5 significant pathways. In T cell, rescinnamine and enalapril share 30 key target genes, such as *CCL2*, *CCL8*, C-X-C Motif Chemokine Ligand 9 (*CXCL9*), *JAK3*, *TNF*, and Lymphocyte Cytosolic Protein 2 (*LCP2*), and all the 6 significant pathways of T cell. The shared target genes and pathways in corresponding cells were previously shown related to death from COVID-19^57–60^.

## Discussion

This study presents a Single-cell Guided pipeline to Aid Repurposing of Drugs (ASGARD) as a new generation of personalized drug recommendation system. To evaluate the accuracy of ASGARD in single drug repurposing, we compared ASGARD to other repurposing methods that utilize bulk cell RNA-Seq (CLUE and DrInsight) or single-cell RNA-Seq data (Alakwaa’s and Guo’s) on a variety of diseases, including breast cancer, leukemia, and COVID-19. ASGARD performs much better than all these methods in predicting drugs/compounds (Figs. 2, 3, Supplementary Fig. 2). The performance of ASGARD is also robust across different sizes and proportions of cell populations, as well as differential expression levels (Supplementary Fig. 3). Moreover, we highlight that ASGARD defines a drug score to summarize drug efficiency across multiple selected cell clusters. These important functions are missing in other simpler single-cell RNA-Seq drug reposition pipelines by Alakwaa and Guo. Both Alakwaa’ and Luo’s pipelines use the CLUE platform, a cloud-based platform developed by the LINCS Center for signature-gene based drug ranking^61^. Additionally, Luo’s method uses log fold change as the additional threshold to filter the gene query. ASGARD on each cluster is related to DrInsight, a concordantly expressed genes (CEG) based, enhanced drug repurposing method compared to other signature-based searching methods^30^. It uses order statistics to directly measure the concordance (e.g. inverse association) between the disease data and drug-perturbed data and identifies concordantly expressed genes (CEGs). CEGS are used as features to further formulate an outlier sum statistic for drug selection, rather than the connectivity score (usually −90) based cut-off for drug selection. The CEG and outlier sum statistic contribute to higher performance in ASGARD.

ASGARD achieves drug ranking for the disease/patient by a drug score that evaluates the treatment efficacy across the user-selected cell clusters (Formula 1 in “Methods” section). The prediction using the multi-cluster drug score shows a significantly (*P*-value < 0.05, Student’s *t* test) better AUC than the prediction based on individual clusters (Figs. 2 and 3). It suggests that targeting an individual cell cluster is not sufficient for successful drug prediction. Instead, targeting multiple essential diseased cell clusters is a more appropriate strategy for drug prediction. On the other hand, it is not ideal to propose drug repurposing using bulk RNA-seq, a mixture of all cells, as done by traditional methods (e.g. CLUE and DrInsight). Significant heterogeneity exists in different T cell populations; not all these cells play equal roles in the diseases^62,63^, reflected by different gene expression responses to drug treatment^64^. ASGARD can distinguish more important T cell types from others and repurpose drugs accordingly, explaining why ASGARD has significantly (*P*-value < 0.05, Student’s *t* test) better AUC performance than traditional bulk methods (Fig. 2b). Moreover, ASGARD also demonstrates variations in drug scores across different patients (Figs. 4b, 5d, and 6d). This result stresses that personalized therapy is necessary for the best therapeutic effect, and utilizing single-cell sequencing information may help to achieve that.

Sparsity and heterogeneity are two major challenges in analyzing single-cell data, and usually cause false discoveries of differentially expressed genes^65^. Previous benchmark study showed that Seurat^18^, DESeq2^26^, edgeR^27^, and Limma^25^ are among the top methods in discovering differentially expressed genes using single-cell data^28^. We here compared the effect of these methods and different parameterization on the downstream drug repurposing, using AUC metric. AUC performance still varies with methods for single-cell differential expression (Fig. 2a, Limma (Bayes) method showed the best average AUC performance compared to all other three methods. Within the Limma method, approaches that model mean-variance with the empirical Bayes approach (Limma Bayes and Limma trend) showed better AUCs than that with the precision weights approach (Limma voom). Similar observations were observed in some of the comparisons in a benchmark study of single-cell differential expression^28^. The empirical Bayes approach is usually more powerful than the precision weights approach when the library sizes are not quite variable between samples^66^. Seurat, a method widely used in single-cell studies, has the 2nd best AUCs in general. In particular, the default mode of Wilcoxon rank-sum test in Seurat has a slightly better average AUC than the t-test and logistic regression (LR) modes. Our comparison revealed that DE methods should be carefully selected according to the status of the dataset to achieve the best performance. Accordingly, ASGARD was designed as a flexible framework supporting various methods for single-cell differential gene expression analysis.

We chose breast cancer or leukemia datasets to illustrate the utilities of ASGARD, given the relative abundance of prior drug knowledge. FDA has approved many drugs predicted by ASGARD, such as neratinib and vinblastine for treating breast cancer^36,37^ (Fig. 4b and Supplementary Fig. 4c), and tretinoin for treating leukemia^51^ (Fig. 5d). Vinblastine and neratinib were predicted for breast cancer in both TNBC patient and PDX datasets. Vinblastine is a vinca alkaloid that has been used in the treatment of metastatic breast cancer since the early 1980s. The regimen of vinblastine/mitomycin is an effective salvage regimen and an excellent first-line chemotherapeutic treatment for women with metastatic breast cancer^41^. Neratinib is a protein kinase inhibitor that was approved in July 2017 as an extended adjuvant therapy in breast cancer^37^. Recently, a randomized phase III clinical trial of 621 patients from 28 countries showed neratinib significantly improved the progression-free survival of patients with advanced breast cancer^67^. Tretinoin, also known as all-trans-retinoic acid (ATRA), is the first candidate predicted by ASGARD for leukemia. Tretinoin targets all significant pathways, such as p53 signaling, cell cycle, and apoptosis pathways, for each diseased cell cluster in leukemia patients (Fig. 5e). These pathways play important roles in the survival of leukemia patients^54^. Consistent with our prediction, tretinoin was approved by the FDA to induce remission in patients with acute leukemia^51^. Tretinoin significantly improves the survival of acute leukemia^68^. Tretinoin with chemotherapy has become the standard treatment for acute leukemia, resulting in cure rates exceeding 80%^69^. The successful prediction of FDA-approved drugs supports the reliability of ASGARD.

Beyond the above described FDA-approved cases, ASGARD also predicts candidate drugs for breast cancer and leukemia. Crizotinib is a candidate from both TNBC patient and PDX model data (Fig. 4b and Supplementary Fig. 4c). Crizotinib is a receptor tyrosine kinase inhibitor that inhibits the growth, migration, and invasion of breast cancer cells in preclinical studies^43,44^. A case report showed the TNBC patient harboring the ALK fusion mutation had a dramatic response to crizotinib treatment^69^. It is also in a phase 2 clinical trial for treating patients with TNBC (ClinicalTrials.gov Identifier: NCT03620643). For leukemia, ASGARD predicts Vorinostat, a histone deacetylase (HDAC) inhibitor, as one of the candidate drugs. Vorinostat was approved by FDR for treating patients with progressive, persistent, or recurrent cutaneous T- cell lymphoma^70^. It induces cell apoptosis in one T cell leukemia cell line in vitro^71^, and improves the outcome of acute T cell lymphoblastic leukemia in animal models^72^. The results of a recent clinical trial (ClinicalTrials.gov Identifier: NCT04467931) show vorinostat is a promising candidate drug for T cell acute lymphoblastic leukemia^73^.

Since ASGARD repurposes candidate drugs to reverse “diseased” cells to “normal” cells, it’s important to set proper controls according to the aim of the study. The best controls are arguably from the normal tissues of the same patients, and the next best ones are from the patients without such a disease but matched on other major confounders. Although consortiums such as Human Cell Atlas aim to obtain normal tissues from clinically healthy samples, it may not be easy to obtain normal samples for some diseases. Under such scenarios, samples from the very early stage of the disease or controls from tissues with the most relevant origin could be substitutes, until the data from the normal tissues are available. Additionally, a recent report has proposed using a deep-learning based approach to identify the best reference control tissue, an attractive strategy that relies much less on prior-assumptions^74^. Additionally, ASGARD built the drug reference using drug responses data from LINCS L1000 project^21^, which were collected from 98 cell lines. We divided the drug reference into several tissue specific drug references according to the tissue origin of the cell lines. It’s highly recommended to select tissue specific drug references when using ASGARD. For example, for drug repurposing in breast cancer, it is best to use drug responses collected from breast cell lines. If there is not a proper cell-type specific reference for the target disease, it might be worth identifying the cell line whose base-line gene expression profiles are most similar to “control” samples, after adjusting for systematic differences between cell line vs. primary tissues (e.g. using transfer learning).

Altogether, this study shows clear evidence that ASGARD defines a single-cell-based reliable drug score for repurposing confident drugs, which were approved or in clinical trials for breast cancer, leukemia, and COVID-19, respectively. It also provides new applications for drugs that warrant further clinical studies. In all, ASGARD is a single-cell guided pipeline with significant potential to recommend repurposeful drugs.

## Methods

### Single-cell RNA sequencing (scRNA-seq) data

We obtained multiple scRNA-seq datasets from the Gene Expression Omnibus (GEO) database. ScRNA-seq data of cells from 4 Triple-Negative-Breast-Cancer (TNBC) patients and 4 healthy controls are from “GSE161529”. Epithelial cells from Patient-Derived Xenografts (PDXs) models of 2 patients with advanced metastatic TNBCs and adult human breast epithelial cells from 3 healthy women are from GEO with accession numbers “GSE123926”^11^ and “GSE113197”^24^, respectively. Another scRNA-seq pediatric bone marrow mononuclear cells (PBMMC) dataset from 2 Pre-T acute lymphoblastic leukemia patients and three healthy controls is from GEO with accession number “GSE132509”^22^. The last set of scRNA-seq datasets are single cells from the bronchoalveolar lavage fluid (BALF) of 15 severe COVID-19 patients (4 deceased and 11 cured) from GEO with accession numbers “GSE145926”^13^ and “GSE158055”^23^.

### Processing of scRNA-seq data

ASGARD accepts processed scRNA-seq data from the Seurat package^18^. In this study, genes identified in fewer than three cells are removed from the dataset. We used the same criteria as in their original studies to filter cells^11,13,24^. Preprocessing steps remove the following cells from the dataset: (1) epithelial cells from breast cancer PDXs and healthy breast tissues with fewer than 200 unique genes, (2) PBMC cells from leukemia patients and healthy controls with fewer than 200 unique genes, and (3) BALF cells from COVID-19 patients with fewer than 200 unique genes, more than 6000 unique genes or have a proportion of mitochondrial genes larger than 10%^13^. For consistency, cells from TNBC patients with fewer than 200 unique genes are also removed from the dataset^39^. We used cell cycle marker genes and linear transformation to scale the expression of each gene and remove the effects of the cell cycle on gene expression.

### Cell pairwise correspondences

ASGARD suggests using functions from Seurat for cell pairwise correspondences. In this study, gene counts for each cell were divided by the total counts for that cell and multiplied by a scaling factor (default is set to 10,000). The count matrix was then transformed by log 2(count+1) in R. To identify gene variance across cells, we first fitted a line to the relationship of log(variance) and log(mean) using local polynomial regression (loess). Then we standardized the feature values using the observed mean and expected variance (given by the fitted line). Gene variance was then calculated on the standardized values. We used the 2000 genes with the highest standardized variance for downstream analysis. Then we identified the K-nearest neighbors (KNNs) between disease and normal cells based on the L2-normalized canonical correlation vectors (CCV). Finally, we built up the pairwise cell correspondences by identifying mutual nearest neighbors^18^.

### Cell clustering and annotation

We applied principal component analysis (PCA) from Seurat on the scaled data to perform the linear dimensional reduction. Then we used a graph-based clustering approach^18^. In this approach, we first constructed a KNN graph based on the euclidean distance in PCA space and refined the edge weights between any two cell pairs using Jaccard similarity. Then we applied the Louvain algorithm of modularity optimization to iteratively group cell pairs together. We further ran non-linear dimensional reduction (UMAP) to place similar cells within the graph-based clusters determined above together in low-dimensional space. To annotate clusters of cells, we ran an automatic annotation of single cells based on similarity to the referenced single-cell panel using the SingleR package^75^. We used the dominant cell type (>50% cells) as the cell type of the cluster.

### Drug repurposing

ASGARD supports importing differentially expressed genes calculated from multiple external methods, including Limma^25^, Seurat (Wilcoxon Rank-Sum test)^18^, DESeq2^26^, and edgeR^27^. The differentially expressed gene list in disease is transformed into a gene rank list. ASGARD uses 21,304 drugs/compounds with response gene expression profiles in 98 cell lines from the LINCS L1000 project^21^. A differential gene expression list in response to drug treatment is also transformed into a gene rank list. ASGARD further identifies potential candidate drugs that yield reversed gene expression patterns from those of diseased vs. normal cells, using the DrInsight package^30^ (version: 0.1.1). Specifically, it identifies consistently differentially expressed genes, which are up-regulated in cells from diseased tissue but down-regulated in cells with drug treatment, or down-regulated in cells from diseased tissue but up-regulated in cells with drug treatment. It then calculates the outlier-sum (OS) statistic^76^, representing the effect of reversed differential gene pattern by the drug treatment. The Kolmogorov–Smirnov test (K-S test) is then applied to the OS statistic, to show the significance level of one drug treatment relative to the background of all other drugs in the dataset. The reference drug dataset contains gene rank lists of 591,697 drug/compound treatments from the LINCS L1000 data, as mentioned above. The Benjamini-Hochberg procedure is used to adjust *P*-values from the K-S test to control False Discovery Rate (FDR) of multiple hypothesis testing^77^.

### Drug evaluation

ASGARD defines a drug score at the individual patient level (Formula 1), which calculates the drug efficacy across all single-cell clusters in a given patient’s scRNA-Seq data. The drug score estimates drug efficacy using the cell type proportion, the significance of reversed differential gene expression pattern (FDR), and the ratio of reversed significantly deregulated genes over disease-related (or selected) single-cell clusters. The drug score is estimated by the following formula:

1 $${Drug}\,{score}=\mathop{\sum }\limits_{k=1}^{n}\left(\frac{{{Num}({Cell})}_{k}}{{Num}({Total}.{Cell})}*\left({-\log }_{10}^{{{FDR}}_{k}}\right)*\frac{{{Num}({ReversedGene})}_{k}}{{Num}({{DiseasedGene}})_{k}}\right)$$

In this formula, $k$ is a particular single-cell cluster, $n$ represents all disease-related (or selected) single-cell clusters, $\frac{{{Num}({Cell})}_{k}}{{Num}({Total}.{Cell})}$ represents cellular proportion of the cluster $k$ in all diseased cells, ${-\log }_{10}^{{{FDR}}_{k}}$ represents the significance of reversed differential gene pattern in the cluster $k$ by drug treatment, and $\frac{{{Num}({ReversedGene})}_{k}}{{Num}({{DiseasedGene}})_{k}}$ represents the ratio of reversed disease-related genes by drug treatment. Specifically, ${Num}({To}{tal}.{Cell})$ is the total number of cells in the sample and ${{Num}({Cell})}_{k}$ is the number of cells in the cluster $k$. ${{FDR}}_{k}$ is the drug’s FDR-adjusted *p*-value (significance of reversed differential gene pattern) for cluster $k$. ${{Num}({DiseasedGenes})}_{k}$ is the number of significantly deregulated genes in a cluster $k$, while ${{Num}({ReversedGenes})}_{k}$ is the number of significantly deregulated genes in a cluster $k$ that can be reversed by the drug. To allow a comparison of drug efficacy across patients, ASGARD also provides a standardized drug score, which has a scale of 0 to 1 (Formula 2).

2 $${Standardized}\,{Drug}\,{Score}\,=\,1-\frac{{Rank}({Drug})}{{Total}\,{Num}({Drug})}$$

Besides the drug score, ASGARD further provides Fisher’s combined *P*-value^78^ over the original *P* value of every cluster. The combined *p*-value is calculated as the right-tail probability ${P}_{{x}^{2}\left(2n\right)}(T\, > \,t)$, where $t=-2{\sum }_{i=1}^{n}{\log }_{10}^{{P}_{i}}$. The BH FDR is used to adjust Fisher’s combined *P*-value. The adjusted Fisher’s combined P-value (FDR) is independent of the drug score. The FDR and drug score can be used together or independently for drug selection. By default, ASGARD uses the drug score for drug selection. Drugs with a higher value of drug score are supposed to have better therapeutic effects than those with a lower value.

### Benchmarking ASGARD

We use the receiver operating characteristic curves (ROCs) and the areas under the ROC curves (AUCs) to compare the performance of ASGARD with those of the other two pipelines, as well as bulk methods. Since these pipelines/ methods report both drugs and compounds, we let ASGARD report both drugs and compounds in the comparisons with other pipelines/methods. ROCs and AUCs are calculated for each pipeline using the pROC package^79^. In ROC and AUC estimation, we regarded FDA-approved drugs and compounds used in advanced clinical trials or have been proven effective in animal models as positive cases (Supplementary Data 4), and all other drugs as negative cases. To identify drugs and compounds used in advanced clinical trials or have been proven effective in animal models, we used three databases that are ClinicalTrials.gov, PubMed, and PubChem, and all the drugs and compounds we found are listed in Supplementary Data 4.

We determined the need to assess the robustness of the three pipelines on different (1) sizes, (2) similarities, and (3) unbalances of single-cell populations. For (1), the Bootstrapping method in R^80^ generated simulation data of different sizes by randomly drawing the same number of disease and normal cells from “GSE123926” and “GSE113197”. For (2), additional simulation data are generated by adjusting the differential gene expression levels from 20% to 90% of the original differential levels of the single-cell cluster, based on “GSE123926” and “GSE113197”. For (3), simulation data is generated by randomly drawing 5000 cells with diseased cell proportions ranging from 20% to 90%, thereby yielding unbalanced populations.

### Drug score analysis

To examine the impact of each cell type on a drug score, we conducted in silico drop-one-out experiments, excluding one cell type from the scRNA-Seq data at a time. The difference between the new drug score and the original drug score is then calculated to reflect the contribution of each cell type to the drug prediction.

To validate ASGARD, we compared it with another drug response prediction method TRANSACT^81^, on the TNBC dataset (“GSE161529”). Since TRANSACT is a method working on bulk gene expression data, we took the mean expression value of each gene across all cells as the pseudo-bulk expression value of that gene. To fit TRANSACT with the dataset we used, we changed two parameters, number_pc[‘target’] and n_pv, to 3 and maintained all other parameters at the same value as the authors’ original report^81^.

### Statistics

All data are presented as mean ± standard deviation (SD), except otherwise stated. *P*-values are adjusted with Benjamini–Hochberg (BH) false discovery rate (FDR). Differences were considered significant when adjusted *P*-value < 0.05. The test used is mentioned in the figure legend.

### Reporting summary

Further information on research design is available in the Nature Portfolio Reporting Summary linked to this article.

## Supplementary information

Supplementary Information

Description of Additional Supplementary Files

Dataset 1

Dataset 2

Dataset 3

Dataset 4

Reporting Summary

## Data Availability

ScRNA-Seq data are available in Gene Expression Omnibus (Accession number: “GSE161529”, “GSE123926”, “GSE113197”, “GSE132509”, “GSE158055”, and “GSE145926”). Phase I LINCS L1000 data are available in Gene Expression Omnibus (Accession number: “GSE92742”). Phase II LINCS L1000 data are available in Gene Expression Omnibus (Accession number “GSE70138”). All other relevant data supporting the key findings of this study are available within the article and its Supplementary Information files or from the corresponding author upon reasonable request. Source data are provided with this paper.

## Source data

Source Data

## Code availability

ASGARD is available as an R package on GitHub (https://github.com/lanagarmire/ASGARD)^82^ under the PolyForm Noncommercial License. The scripts used in this study are available on GitHub (https://github.com/lanagarmire/Single-cell-drug-repositioning)^83^.
