# Supplementary material for: ASGARD is A Single-cell Guided Pipeline to Aid Repurposing of Drugs
Source: Nat Commun. 2023 Feb 22;14:993. doi: 10.1038/s41467-023-36637-3 (PMC9945835; doi:10.1038/s41467-023-36637-3)
Supplement: Supplementary file 1 — Supplementary Information [file 41467_2023_36637_MOESM1_ESM.pdf]

Supplementary Information

Supplementary Figures

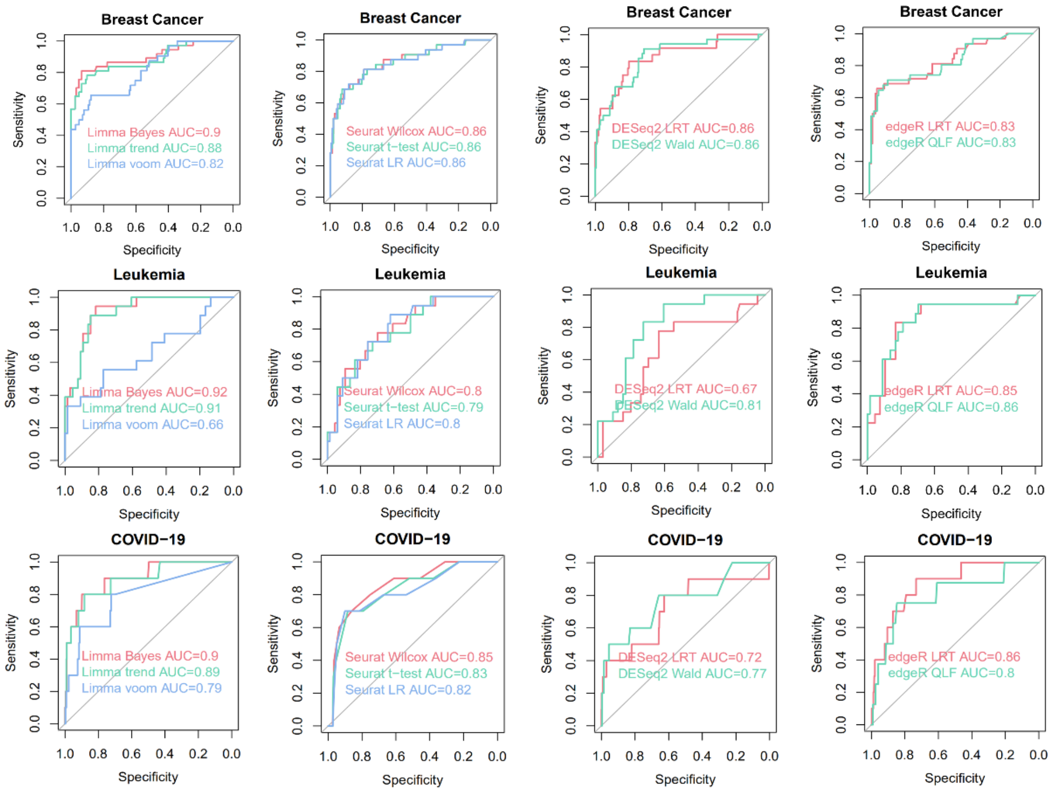

Supplementary Figure 1. The impact of single-cell differential expression methods on ASGARD performances. The receiver operating characteristic (ROC) curves and area under curve (AUC) scores of the ASGARD, using DE analysis methods (Limma, DESeq2, Seurat, and edgeR) with tuned parameters. The tests are done on advanced metastatic breast cancer, acute lymphoblastic leukemia, and coronavirus disease 2019 (COVID-19), respectively. For each method with specific parameter, the ROC curve and AUC value are present in the same color. Source data are provided as a Source Data file.

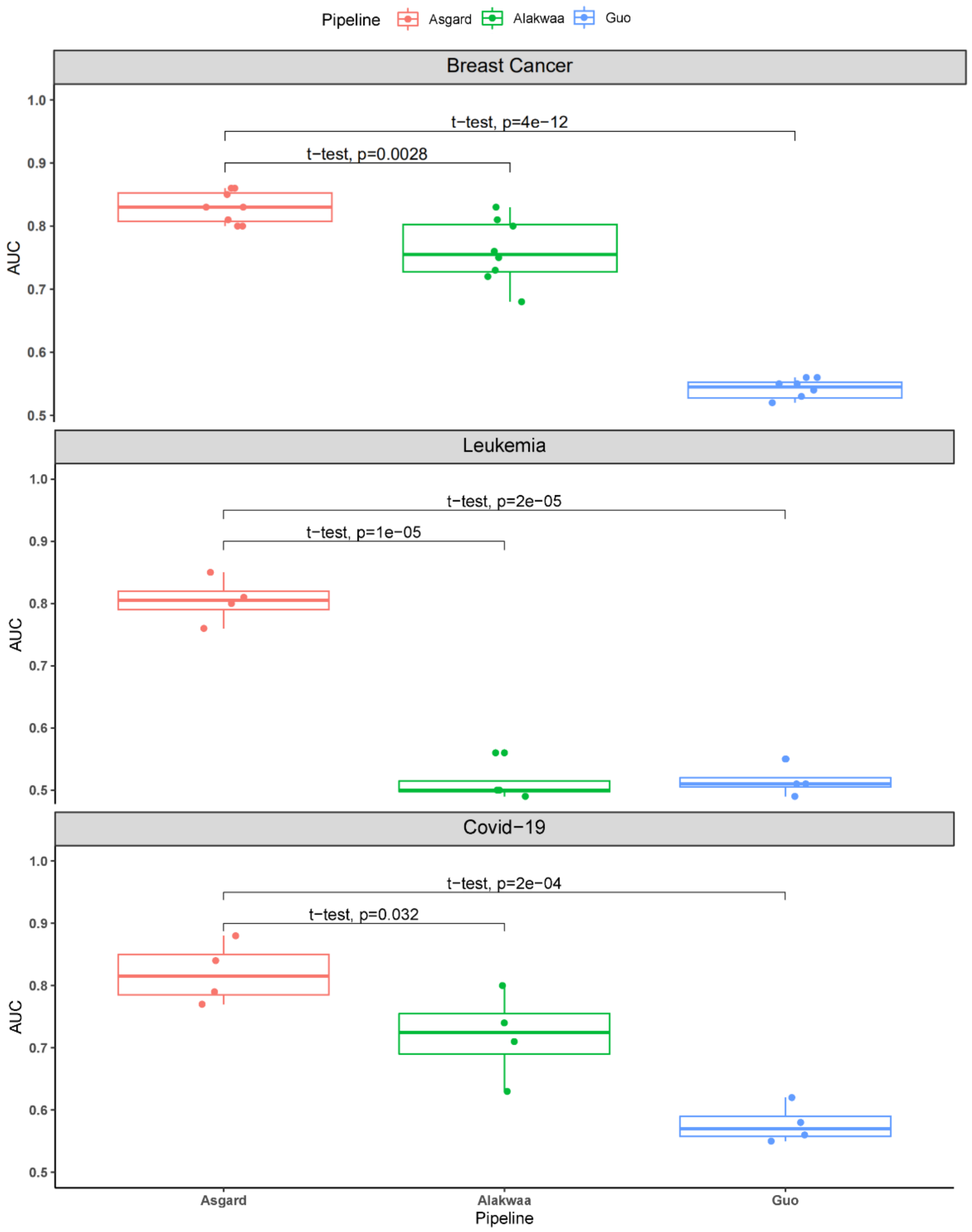

Supplementary Figure 2. Boxplots on AUCs of the three single-cell-based repurposing pipelines, based on all cell clusters, as shown in Figure 3. Lower and upper hinges of boxes represent 25th to 75th percentiles of AUC values. The central lines of boxes represent the median AUC values. The whiskers of boxes extend to highest and lowest AUC values no greater than 1.5× interquartile range. For breast cancer, n=8 independent single-cell clusters. For leukemia, n=4 independent single-cell clusters. For COVID-19, n=4 independent single-cell clusters. The P-values were determined by two-sided t-test. Colors: Red, Asgard; Green, Alakwaa's pipeline; Blue, Guo's pipeline. Source data are provided as a Source Data file.

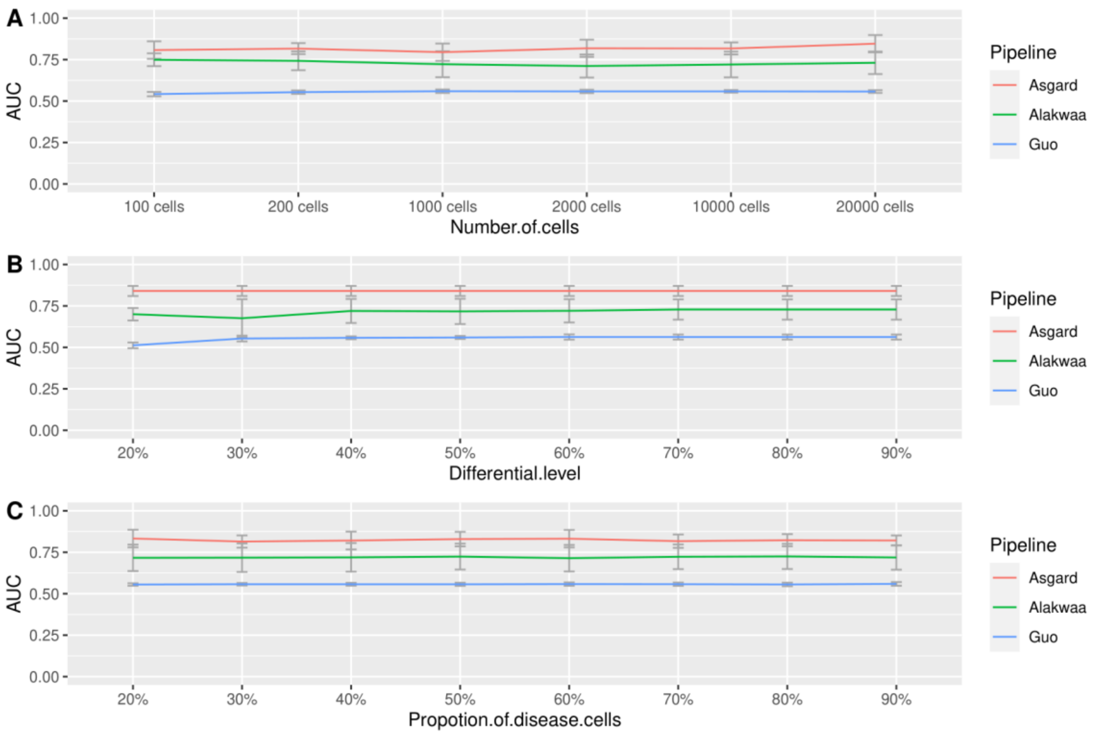

Supplementary Figure 3. AUC scores of the three single-cell-based repurposing pipelines, using simulation data adapted from the real datasets. (A) The effect of varying total cell sizes from 100 cells to 20000 cells. **(B)** The effect of varying differential expression levels from 20% to 90%. **(C)** The effect of varying the proportions of diseased cells ranges from 20% to 90%. Data are presented as mean values +/- SD. For A-C, n=3 independent experiments. Colors: Red, Asgard; Green, Alakwaa's pipeline; Blue, Guo's pipeline. Source data are provided as a Source Data file.

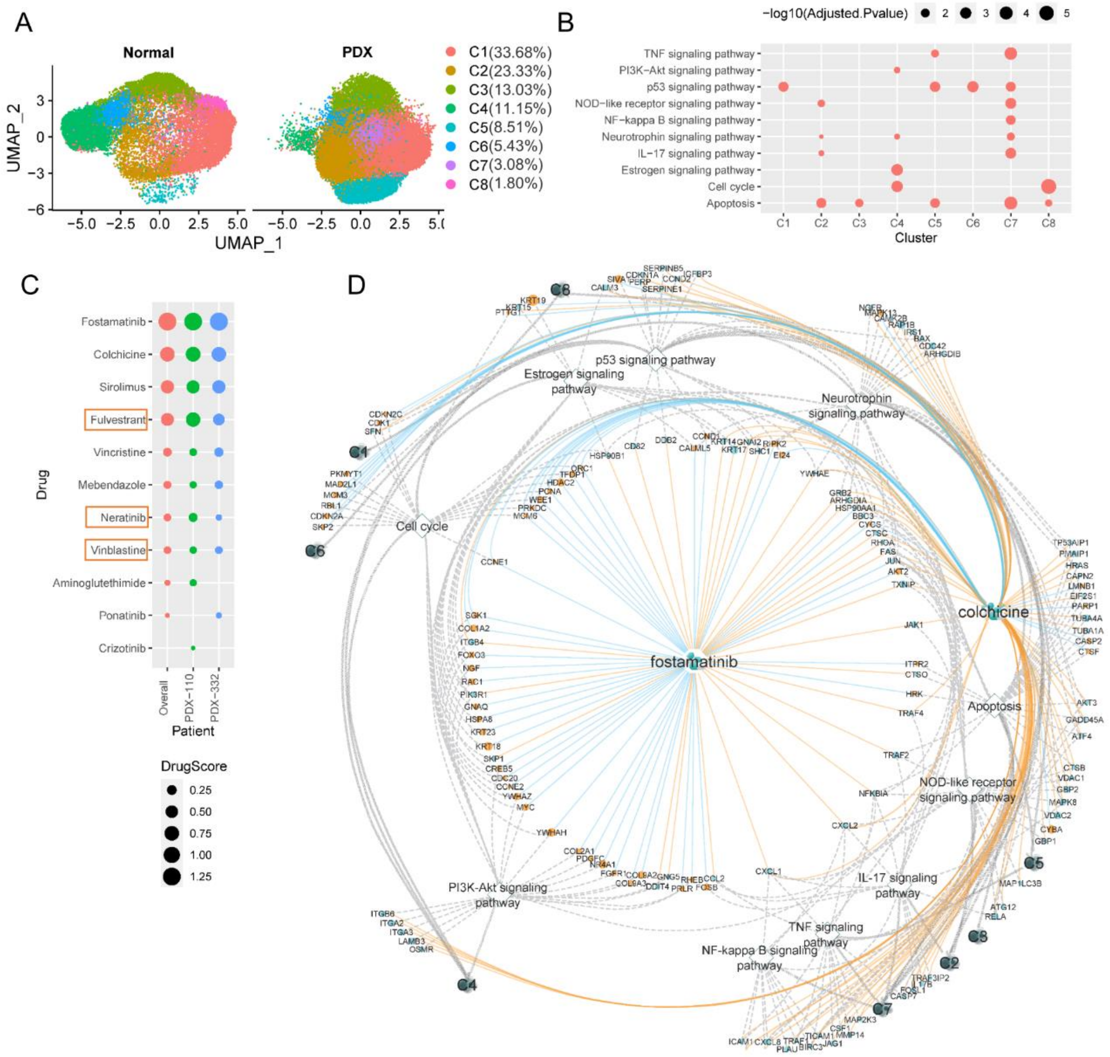

Supplementary Figure 4. Drug repurposing in Patient-Derived Xenograft (PDX) models derived from advanced metastatic TNBC patients. (A) UMAP plots of single-cell data from 3 normal controls and 2 breast cancer PDX samples. **(B)** Pathway enrichment analysis (breast cancer vs normal) for each single-cell cluster. P-values were determined by two-sided Fisher’s exact test and were adjusted by BH FDR. **(C)** The overall drug score combining both PDX models and drug score in each breast cancer PDX model, among top-ranked significant single drugs (FDR<0.05). Drugs approved for breast cancer treatment by the FDA are labeled in red boxes. **(D)** The drug candidates fostamatinib and colchicine, their target genes, pathways, and single-cell clusters. All labels and their annotations are the same as Figure 4F. Source data are provided as a Source Data file.
