# Supplementary material for: ASGARD is A Single-cell Guided Pipeline to Aid Repurposing of Drugs
Source: Nat Commun. 2023 Feb 22;14:993. doi: 10.1038/s41467-023-36637-3 (PMC9945835; doi:10.1038/s41467-023-36637-3)
Supplement: Supplementary file 2 — Description of Additional Supplementary Files [file 41467_2023_36637_MOESM2_ESM.pdf]

<u>**Description of Additional Supplementary Files**</u>

Supplementary Data 1. Predicted drugs for breast cancer PDX model. P-values were determined by two-sided K-S test and were adjusted by BH FDR.

Supplementary Data 2. Predicted drugs for leukemia. P-values were determined by two-sided K-S test and were adjusted by BH FDR.

Supplementary Data 3. Predicted drugs for COVID-19. P-values were determined by two-sided K-S test and were adjusted by BH FDR.

Supplementary Data 4. FDA-approved drugs and compounds used in advanced clinical trials or have been proven effective in animal models
